# Synesthesia and release phenomena in sensory and motor grounding. Cases of disinhibited embodiment?

**DOI:** 10.3389/fpsyg.2015.00054

**Published:** 2015-01-30

**Authors:** Brian F. Gray, Julia Simner

**Affiliations:** ^1^Department of Psychology, University of EdinburghEdinburgh, UK; ^2^School of Psychology, University of SussexBrighton, UK

**Keywords:** synesthesia, embodiment, embodied cognition, grounding, perception-cognition interface, motor release, alien hand sign, anarchic hand syndrome

*Synesthesia* is an unusual condition occurring in at least 4% of the population (Simner et al., 2006) in which certain stimuli trigger unusual perceptions which the physical properties of the stimulus alone are not sufficient to account for. For instance, in *grapheme-color synesthesia*, the sight of black-and-white printed letters or numbers triggers the experience of consistent and specific colors (e.g., A might be red, 7 yellow: Smilek et al., 2001). The associations reported by any given synesthete tend to be remarkably consistent over time (e.g., Simner et al., 2005). Hence if A is red for a certain synesthete, it will tend to always be red. There is evidence for a genetic component to the condition (Ward and Simner, 2005; Asher et al., 2009; Tomson et al., 2011), and evidence too that the brains of synesthetes are structurally and functionally different to those of non-synesthetes. For example, in a *sound-color* and *sound-taste synesthete*, Hänggi et al. (2008) found altered patters of brain volume and functional anisotrophy (FA) values (indicative of greater white matter coherence). These included increased FA values in primary auditory cortex, as well as increased gray and white matter volumes in the same region, and also structural differences in gray and white matter in visual and gustatory regions, i.e., increases in occipital regions, and increases and decreases in insular cortex (for review see Hubbard, 2012; Rouw, 2012).

It has often been suggested than synesthesia may reveal something about normal cognition (e.g., Cohen Kadosh and Henik, 2007; Wilson, 2012). Ramachandran and Hubbard (2001) have even suggested that the origins of human language may lie in synesthetic-like correspondences between speech sounds and the physical properties of objects they refer to (see Cuskley and Kirby, 2013 for review). These theories usually describe synesthesia as a “blending of the senses,” in which an experience in one *sensory* modality triggers an experience in another *sensory* modality; for example sounds triggering colors. However, synesthesia is not always, or even usually, cross-sensory: the most common variants are in fact triggered by language (e.g., colors from graphemes or words; Simner et al., 2006). In such cases, the trigger is a cognitive (linguistic) construct rather than sensory percept (for related discussions see Simner, 2007 and Mroczko-Wąsowicz and Nikolić, 2014). Indeed, many types of synesthesia involve conceptual elements—often linguistic—and we will argue that these variants may reveal information about the interface between perception and cognition.

One example of how synesthesia can link cognition and perception comes in the variant known as lexical-gustatory synesthesia. In this, tastes are experienced in the mouth when synesthetes hear, speak, read, or think about words (Ward and Simner, 2003). Simner and Ward (2006) showed that these sensations are tied to word lemmas, the abstract semantic/syntactic memory traces of words that are distinct from purely visual and acoustic information about the word-form. Given this, we might conclude that the perceptual taste sensations of these synesthetes appear to be triggered by truly abstract cognitive thought, as a case of direct interaction between cognition and perception. Our proposal here begins with the suggestion (also made by others, see below) that these kinds of interactions may occur in all people, and that synesthetes may simply differ primarily in their conscious awareness of them.

Our proposal focusses particularly on theories of embodied cognition. These theories contend that conceptual thought and language are ‘embodied’ or ‘grounded’ in neural systems for perception and action (e.g., Glenberg and Kaschak, 2002; Fischer and Zwaan, 2008). Hence, hearing a word or thinking about a concept would trigger a “simulation” of stored perceptual or motor information (Barsalou, 2003; Glenberg and Gallese, 2012). The taste of lemon, for instance, would form part of the semantics of the word “lemon,” and the implicit mental activation of that taste would be linked to its phonological representation. Whereas in most people these perceptual connections are implicit, our hypothesis, first suggested by Simner and Ward (2006), is that in lexical-gustatory synesthetes for example, these same simulations somehow attain conscious awareness, so that hearing the word “lemon” actually evokes the taste.

We suggest therefore that normal “embodied” links between perceptions (taste of lemon) and words (“lemon”) may somehow be consciously experienced by synesthetes, due to some type of disinhibited or over-exuberant activation which is normally subdued in the average person. Of course, this proposal would only initially account for words that have an inherent taste, color, or the like (e.g., “lemon”). The problem however is that for many synesthetes, almost *all words* (e.g., “man,” “house,” “reach,” “and”) induce tastes, often without any obvious connection to their meaning at all (e.g., “reach” might taste of fruit sweets). How might this be explained and how could such widespread tastes link back to the idea of embodiment? Importantly, Simner and Haywood (2009) have shown that, in the case of lexical-gustatory synesthesia, the first words to acquire tastes in childhood synesthesia are very likely to have been food words, which acquire tastes in a semantically-direct way—so “apple” tastes of apple, “peach” tastes of peach (see also Ward et al., 2005). Simner and Haywood suggest that these tastes, or variants thereof, then spread outwards to phonologically similar words that do not have tastes of their own (e.g., “beach” tastes of peach; “reach” tastes of peach-flavored fruit sweets; Simner and Haywood, 2009). Put differently, synesthetic tastes appear to spread throughout the lexicon along the same connections that facilitate phonological priming effects: hearing “beach” might activate the word “peach,” and thereby acquire the taste of peach. The more often this word is heard and the taste experienced, the more strongly the two would become connected, so that before long, synesthetes will differ from non-synesthetes in not only having conscious tastes for food words, but also in having tastes for other words in their mental lexicon too^1^.

One little-known phenomenon that provides additional evidence for a link between synesthesia and embodied cognition is *vision-touch synesthesia* (Blakemore et al., 2005), in which people report a physical sensation of being touched themselves when they observe others being touched. It has also been called *mirror-touch synesthesia* by analogy with the “mirror neurons” found initially in the monkey premotor cortex, and hypothesized to exist also in humans. These fire both when performing a certain action and when seeing that action performed (see Rizzolatti and Craighero, 2004, for a review). “Mirror-touch” is a particularly illuminating example of synesthesia, because the underlying connectivity implicated—the mirror neuron system—is not something assumed to be peculiar to these synesthetes, but is hypothesized to play a key role in the imitation, prediction, and understanding of others' behavior in the general population (e.g., Wilson and Knoblich, 2005). The embodied simulation theory of social cognition, for example, proposes that the mirror system, by internally “simulating” the motor and somatosensory states of conspecifics, allows us to understand them (Gallese and Sinigaglia, 2011). Indeed, mirror-touch synesthetes have been found to have greater empathy than controls, which has been interpreted as evidence that mirror neurons may indeed play such a role (Banissy and Ward, 2007). Banissy and colleagues have therefore argued that the normally implicit somatosensory stimulation triggered by observing others might be experienced in an extreme form in mirror-touch synesthetes^2^. This view is highly compatible with our own, which also raises a parallel case for other forms of synesthesia (see also Simner and Ward, 2006).

So far, we have argued that the connections between perception and cognition seen in synesthesia might be present in all people, but inhibited in the general population, consistent with embodied theories of language and cognition. However, these theories are in fact best-known as they apply to action: i.e., that action concepts (and the semantics of action words) are also embodied, this time in the motor system. This widespread view of embodiment suggests that perceiving and understanding action words (e.g., “hit”) or indeed observing the actions of others, involves mentally simulating them. So if synesthesia is the result of disinhibited simulation in the sensory system, what would be the result of disinhibition of embodied simulation in the *motor* system? (Our hypothesis thus far certainly predicts there might be such a phenomenon.) In response we point to candidates from the range of behaviors known as “motor release phenomena.”

Motor release phenomena are a variety of syndromes involving automatic motor behaviors, most commonly seen following damage to the frontal lobes from stroke, but also observed in other populations with known or suspected dysfunction of frontal control systems. These include patients with various psychiatric illnesses and children with attention deficit hyperactivity disorder (Archibald et al., 2001). They can be broadly classified into three types (Lhermitte, 1983): *disinhibition of basic reflexes* such as manual groping, when the patient's outstretched hand will follow an object being moved; *imitation behavior*, in which patients automatically and unwillingly find themselves copying observed actions; and *utilization behavior*, where patients compulsively pick up objects placed in their view, and either toy with them or use them for their intended purpose. These behaviors are perhaps most striking when they only affect one side of the body, such that one arm appears to act independently of its owner's will, known as the famous “alien hand sign” or “anarchic hand syndrome” (e.g., Goldberg and Bloom, 1990).

All these phenomena are thought to arise from the disinhibition of circuits which link perceptual input to motor output, possibly through the mirror neuron system (Berthier et al., 2006; McBride et al., 2013). Evidence from masked priming tasks supports the view that these links are automatically and unconsciously inhibited in healthy people (e.g., McBride et al., 2013). Crucially for theories of embodied cognition, there is evidence that this perception-action link is conceptually or linguistically mediated. Goldberg and Bloom (1990) reported several cases of alien hand sign where the alien hand performed actions that were merely mentioned by the investigator. Schaefer et al. (2013), too, report a patient who would pick up objects with her alien hand when told to by the experimenter, but was unable to do so of her own volition. Furthermore, some patients could exercise some control over their alien hand by “talking to it”: when their alien hand gripped an object, they were able to get it to release by telling it to “let go.” These observations fit well with the predictions of embodied cognition: if understanding sentences that refer to actions involves their simulation by the motor system, then disinhibition of these simulations would indeed lead to the actions described being overtly performed (McBride et al., 2013).

As noted above, theories of embodied cognition propose that the brain's systems for perception and action are used in cognition. We have suggested that both types of simulation can be disinhibited in some people, resulting in synesthesia in the case of the sensory system (see also Simner and Ward, 2006), and release phenomena in the case of the motor system (see also McBride et al., 2013). The question might be asked why the two types of disinhibition are not more analogous: why, for instance, do we not see cases of synesthesia often arising as a result of stroke? Our first answer is that the types of inhibition involved are somewhat different. In the case of motor embodiment, it is suggested that a motor plan is initiated, but not executed. In sensory embodiment, on the other hand, perceptual information is activated, but does not, in most people, reach conscious perceptual awareness. These two forms of “inhibition” are doubtlessly underpinned by very different mechanisms. Secondly, there are in fact cases of synesthesia-like experiences occurring in non-synesthetes following brain damage (e.g., Ro et al., 2007; Brogaard et al., 2013). There are also cases where it emerged after the patient became blind late in life (Armel and Ramachandran, 1999), or under the influence of hallucinogens (see Luke and Terhune, 2013, for review), or after long experience with meditation (Walsh, 2005). They have even been experimentally induced by post-hypnotic suggestion (Cohen Kadosh et al., 2009). While there is disagreement as to the extent to which developmental, acquired, and other forms of synesthesia might share common mechanisms (Sinke et al., 2012, but cf. Brogaard, 2013), these examples nonetheless support the hypothesis that the cognition-perception links seen in synesthesia may exist in some inhibited form in all people, and may become “released” following trauma, disease or unusual environmental interactions.

In summary, motor release phenomena have been interpreted as evidence that the motor system is involved when observing and describing actions, and in object affordances (e.g., McBride et al., 2013). We have proposed that, similarly, some types of synesthesia suggest that the *sensory* system, too, may play a role in language and conceptual thought. We have therefore proposed a relationship between synesthesia and release phenomena, in that each may be considered in terms of disinhibited embodiment in sensory and motor systems respectively. Overall, our arguments suggest that synesthesia may represent a case *par excellence* of the cognition-perception interface, showing an outward perceptual manifestation of implicit associations that lie at the heart of embodied cognition.

## Conflict of interest statement

The authors declare that the research was conducted in the absence of any commercial or financial relationships that could be construed as a potential conflict of interest.

## Footnotes

^1^Our account best fits variants of synaesthesia which, like embodiment, are influenced by word meanings (e.g., lexical-gustatory synaesthesia). Other variants such as grapheme-color synaesthesia may be less relevant to our model because colors are driven by sublexical letter units: since letters have no semantic content they cannot obviously show embodiment effects—at least not on the surface. Nonetheless, embodiment might yet hold sway even in these variants. For example, in grapheme-color synaesthesia, words become colored by their initial letter (e.g., “orate” would be white if the letter “o” is white) but some words such as color-terms are semantically colored (“orange” is often colored orange). This follows our hypothesis, and suggests that embodiment might interact with other mechanisms even in variants of synaesthesia that would seem otherwise unsusceptible to its influences.

^2^A link between synaesthesia and the mirror neuron system has also been made in a different way by Mroczko-Wąsowicz and Werning (2012). They describe cases of synaesthesia where colors are experienced both from movement and from observing/imagining the movement of others. They link this to mirror systems as a way to best model the inducer, and they also discuss this with reference to sensori-motor contingencies (see also Seth, 2014).
